# The effects of volitional hyperpnea on biomarkers of respiratory muscle damage in healthy young men

**DOI:** 10.14814/phy2.70277

**Published:** 2025-03-20

**Authors:** Muneeb Iqbal, Edward Bliss, Eliza J. Whiteside, Ben Hoffman, Dean E. Mills

**Affiliations:** ^1^ School of Health and Medical Sciences, University of Southern Queensland Ipswich Queensland Australia; ^2^ Respiratory and Exercise Physiology Research Group School of Health and Medical Sciences, University of Southern Queensland Ipswich Queensland Australia; ^3^ Centre for Health Research Institute for Resilient Regions, University of Southern Queensland Ipswich Queensland Australia; ^4^ Centre for Future Materials University of Southern Queensland Toowoomba Queensland Australia

**Keywords:** biomarkers, respiratory muscle damage, skeletal troponin I, volitional hyperpnea

## Abstract

High‐intensity exercise hyperpnea places substantial demands upon the respiratory muscles, but whether this causes respiratory muscle damage is unknown. We investigated respiratory muscle damage following volitional hyperpnea (equivalent to 85% of participants maximum minute ventilation produced during a maximal incremental cycling test) using a skeletal muscle damage biomarker panel. Eight healthy men (33 + 2 years) underwent 10‐min trials of volitional hyperpnea and rest (control) two weeks apart. Serum was collected before and at 1, 24, and 48 h after both volitional hyperpnea and control trials. Creatine kinase muscle‐type (CKM), fast skeletal troponin I (sTnI) and slow sTnI were measured using enzyme‐linked immunosorbent assay. Two‐way analysis of variance revealed time × trial interaction effects for slow sTnI (*p* = 0.018), but not for CKM (*p* = 0.072) and fast sTnI (*p* = 0.140). Slow sTnI was significantly higher at +24 h post volitional hyperpnea (*p* < 0.001) as compared to the same time point of the control trial. These results indicate that high‐intensity exercise hyperpnea may induce a small amount of respiratory muscle damage as evidenced by the increases in slow sTnI. Future studies including more time points, different respiratory muscle exercise protocols, and examining the differences between sexes could provide additional insights into the utility of blood biomarkers for identifying respiratory muscle damage.

## Graphical abstract

Biomarkers of respiratory muscle damage following volitional hyperpnea.

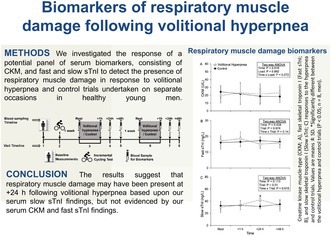

## 1 INTRODUCTION

High‐intensity exercise hyperpnea places substantial demands upon the respiratory muscles as breathing frequency, tidal volume, and the work of breathing increase (Sheel & Romer, 2012). Prolonged and strenuous exercise hyperpnea can also result in diaphragm fatigue (Mador et al., 1996). In peripheral limbs, skeletal muscle damage can also occur following unaccustomed, high‐intensity exercise (Peake et al., 2017). However, there is limited information available about respiratory muscle damage compared to peripheral skeletal muscle damage (Brancaccio et al., 2010; Koch et al., 2014). One of the main factors contributing to this is the challenges associated with certain methodologies that are often employed to analyze peripheral muscle damage. Muscle biopsies, for example, can directly measure the extent of damaged fibers in peripheral skeletal muscle, but this technique has only been applied for the respiratory muscles with small tissue samples collected from patients having abdominal or thoracic surgery (Macgowan et al., 2001; Orozco‐Levi et al., 2001). Although this method provides definitive evidence of damage to the muscle's structure, biopsies are invasive and difficult to sample on most respiratory muscles because of their internal location (van den Berg et al., 2017). One potential method for identifying damage to muscles without the need for invasive procedures involves the use of several imaging techniques, such as computed tomography scans, magnetic resonance imaging, and magnetic resonance spectroscopy. However, the utilization of these imaging techniques is accompanied by high costs, limited accessibility, and results that may also vary depending upon the particular technique used (Hatabu et al., 2020; Tuinman et al., 2020). An alternative method for detecting muscular damage in the respiratory muscles is the utilization of blood‐based biomarkers (Foster et al., 2012). Indirect evidence of structural damage, potentially caused by exercise or overactivity of the respiratory muscles in specific respiratory disorders, could be inferred from the presence or increase in the concentration of blood‐based biomarkers after exercise or respiratory muscle work (Brancaccio et al., 2010; Iqbal et al., 2023). Utilizing blood markers is an easy, cost‐effective, and less invasive procedure compared with anatomical pathology examination that may offer a more efficient and advantageous approach to detecting respiratory muscle damage (Brancaccio et al., 2010; Foster et al., 2012).

Iqbal et al. (2023) and Foster et al. (2012) have found that blood‐based biomarkers of respiratory muscle damage increase following inspiratory pressure‐threshold loading (ITL) in healthy young men. Foster et al. (2012) measured skeletal troponin‐I (sTnI) following 60 min of ITL. sTnI is a regulatory protein that plays an essential role in the contraction of skeletal muscles (Bowman & Lindert, 2019; Gresslien & Agewall, 2016). sTnI is an ideal biomarker for muscle damage due to its specificity to skeletal muscle tissue, quantitative measurability, and sensitivity, making it an accurate indicator of the extent of muscle damage when detected in the bloodstream (Goldstein, 2017; Iqbal et al., 2023; Rasmussen & Jin, 2021). Foster et al. (2012) observed that fast sTnI increased at 1 h (+24%) and 3 days (+72%) post ITL. Slow sTnI was elevated by 24% 4 days post ITL, but there was no change in creatine kinase. Iqbal et al. (2023) observed that creatine kinase muscle‐type (CKM) and fast sTnI increased immediately (+1 h) following ITL, while CKM and slow sTnI increased at +24 and +48 h (Iqbal et al., 2023). This finding suggests that CKM and fast sTnI could be used to assess respiratory muscle damage immediately, while CKM and slow sTnI could be used to assess respiratory muscle damage in the days following conditions that elevate inspiratory muscle work (Iqbal et al., 2023).

We used ITL as a tightly controlled experimental approach that allowed both primary and accessory respiratory muscles to be exercised without peripheral muscle involvement. This technique elevates inspiratory muscle work in individuals while at rest and thus isolates the respiratory muscles in such a way that any changes in systemic blood biomarkers can be assumed to originate from the respiratory muscles. Another experimental approach that also allows respiratory muscles to be exercised without peripheral muscle involvement is volitional hyperpnea performed at rest (Mills et al., 2013). This technique involves participants mimicking at rest the breathing pattern they adopted during a prior exercise bout. As such, this experimental technique results in a breathing and respiratory muscle recruitment pattern that is more physiologically relevant to exercise hyperpnea. However, the effects of volitional hyperpnea on blood‐based biomarkers of respiratory muscle damage have not been tested.

Accordingly, the aim of this study was to investigate the effects of volitional hyperpnea on blood‐based biomarkers of respiratory muscle damage, including CKM and fast and slow sTnI. We hypothesized that CKM, fast and slow sTnI would increase following volitional hyperpnea compared to a control trial.

## 2 METHODS

### 2.1 Participants

Eight apparently healthy young men with respiratory function within normal limits volunteered to participate in the study (Table 1). The exclusion criteria were current cigarette smokers; a history or current symptoms of cardiopulmonary disease; contraindications to exercise testing; and a body mass index of <18.5 or >30 kg/m^2^. A self‐reporting medical history questionnaire confirmed that participants were free from illness and injury and not taking any medication and/or dietary supplements during the study. All participants provided written, informed consent. All study procedures were approved by the University of Southern Queensland Human Research Ethics Committee (H20REA151), which adheres to the Declaration of Helsinki with the exception of registration in a database.

**TABLE 1:** Participant anthropometrics, respiratory function, and maximal exercise capacity. Values are mean ± SD.

Age (years)	33 ± 2
Height (cm)	176 ± 6
Body mass (kg)	83 ± 9
Body mass index (kg/m^2^)	26.5 ± 2.5
FVC (L)	4.63 ± 0.88
FVC (% predicted)	92 ± 17
FEV_1_ (L)	3.69 ± 0.44
FEV_1_ (% predicted)	89 ± 12
FEV_1_/FVC (%)	74 ± 7
FEV_1_/FVC (% predicted)	98 ± 9
P_Imax_ (cmH_2_O)	113 ± 4
P_Imax_ (% predicted)	103 ± 13
P_di_max (cmH_2_O)	72 ± 20
V̇O_2max_ (mL/kg/min)	45 ± 9
V̇_Emax_ (L/min)	140 ± 4

*Note*: Predicted values for pulmonary volumes and capacities are from Quanjer et al. (2012) and for P_Imax_ are from Wilson et al. (1984).

Abbreviations: FEV_1_, forced expiratory volume in 1 s; FVC, forced vital capacity; P_dimax_, maximal transdiaphragmatic pressure; P_Imax_, maximal inspiratory mouth pressure; V̇_Emax_, maximal minute ventilation; V̇O_2max_, maximal oxygen uptake.

### 2.2 Experimental design

**FIGURE 1:**
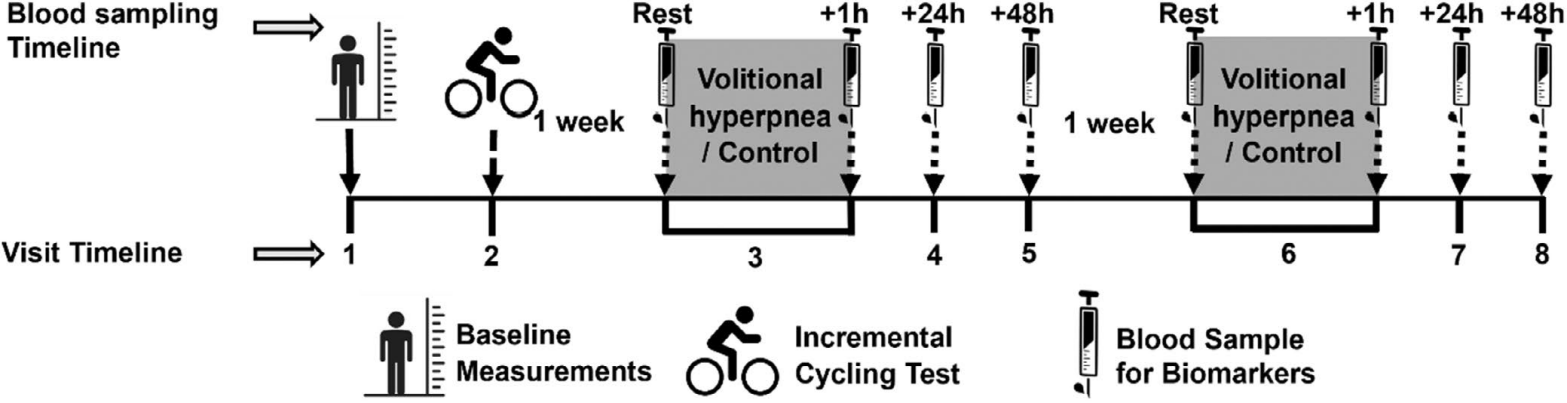
Schematic of experimental design.

Participants attended the laboratory for eight visits on separate days (Figure 1). During Visit 1, height, body mass, pulmonary function, and maximal inspiratory mouth pressure were assessed according to published guidelines and statements (Laveneziana et al., 2019; Miller et al., 2005). Participants were also familiarized with all the study procedures. During Visit 2, participants performed a maximal incremental cycling test. During Visits 3 and 6, participants randomly performed either 10 min of volitional hyperpnea whilst seated on a cycle ergometer at rest or adopted the same position seated on the cycle ergometer and did not perform volitional hyperpnea (control). Blood samples were collected from participants and stored at −80°C for subsequent analysis of respiratory muscle damage biomarkers at rest and at +1 h, +24 h (Visits 4 and 7), and +48 h (Visits 5 and 8) post‐volitional hyperpnea and control trials. Visits 2 (maximal incremental cycling test), 5 (+48 h after volitional hyperpnea or control), and 6 (volitional hyperpnea or control) were separated by at least 1 week. To avoid the effects of the preceding trial, a gap of at least 1 week was provided between the maximal incremental cycling test (Visit 2) and the first experimental condition (either volitional hyperpnea or control; Visit 3). In addition, a gap of 1 week was provided between the day of the last blood sample for the first experimental condition (+48 h; Visit 5) and the second experimental condition (either volitional hyperpnea or control; Visit 6). Participants were required to refrain from moderate‐vigorous exercise for at least 2 days prior to Visits 2, 3, and 6 and to not exercise in the 48 h after each visit. Participants were also instructed to abstain from food (4 h), caffeine (12 h), and alcohol (24 h) before laboratory visits.

### 2.3 Anthropometrical measures and respiratory function

Height and body mass were recorded using a wall‐mounted electronic stadiometer (Seca 213; Seca, Hamburg, Germany) and an electronic scale (Tanita BC‐541; Tanita, Kewdale, Australia), respectively. Pulmonary function was assessed using a spirometer (JAEGER® Vyntus; CareFusion, San Diego, CA, USA) according to published guidelines (Miller et al., 2005). A hand‐held mouth pressure meter (MicroRPM; CareFusion, Frenchs Forest, Australia) was used to measure maximal inspiratory mouth pressure as an index of global inspiratory muscle strength. The maneuver was performed while seated, initiated from residual volume sustained for a minimum of 1 s. The maneuver was repeated until three sequential measurements differing by no more than ±10% or ±10 cmH_2_O, whichever is smallest. The greatest value is recorded for later analysis (Mills et al., 2013).

### 2.4 Maximal incremental cycling test

Participants undertook a maximal incremental exercise cycling test to exhaustion on an electronically braked cycle ergometer (Corival; Lode, Groningen, the Netherlands). Each test began with 5 min of rest and cycling then began at 0 W and power was subsequently increased by 20 W every 30 s in order to result in exercise intolerance within 8–12 min. Participants maintained a constant self‐selected cadence above 60 revs/min. Exercise ceased at the limit of tolerance or when cycling cadence could not be maintained above 60 revs/min. Participants wore a facemask (Model 7940; Hans Rudolph, Shawnee Mission, KS, USA) which was tightly fitted to minimize leaks and connected to a turbine flow sensor (Digital volume transducer; Vyaire Medical, Chicago, IL, USA) that was calibrated using a 3 L syringe. Pulmonary gas exchange was measured breath by breath using a metabolic cart (Vmax® Encore PFT system; Vyaire Medical, Chicago, IL, USA). The highest oxygen uptake (V̇O_2_) and minute ventilation (V̇_E_) recorded in any 30 s period was defined as V̇O_2max_ and V̇_Emax_, respectively.

### 2.5 Volitional hyperpnea

Participants performed volitional hyperpnea at rest whilst seated on the cycle ergometer in a body position identical to that adopted during the maximal incremental cycling test. Participants mimicked the breathing (tidal volume, breathing frequency and duty cycle) and respiratory muscle recruitment (transdiaphragmatic pressure; P_di_) patterns in a square wave manner to a level equivalent to those at 85% of their V̇_Emax_ produced during the maximal incremental cycling test. Our pilot work and others showed that this was the maximal exercise breathing pattern that could be maintained for 10 min (Brown et al., 2008). An audio metronome paced breathing frequency and duty cycle and real‐time visual feedback of tidal volume and P_di_ was provided throughout the test. Isocapnia was maintained during volitional hyperpnea by adding carbon dioxide into the inspiratory circuit in order to maintain resting arterial carbon dioxide partial pressures.

### 2.6 Respiratory pressures

Respiratory muscle work was quantified by measuring esophageal pressure (P_e_) and gastric pressure using two 10 cm balloon‐tipped latex catheters (Model 47–9005; Ackrad Laboratories, Cranford, NJ, USA) attached to two different pressure transducers (MLT844; AD Instruments, Dunedin, New Zealand). Co‐Phenylcaine Forte Spray (Lignocaine Hydrochloride 5%; Phenylephrine Hydrochloride 0.5%; ENT Technologies Pty Ltd., Hawthorne, Australia) was used to initially anesthetize the nasal passage. Then, catheters were passed peri‐nasally into the lower third of the esophagus and stomach, respectively. During the first experimental trial, the distance from the tip of the nares to the most distal point of the catheters was recorded and replicated in the subsequent trial. The esophageal and gastric balloons were filled with 1 and 2 mL of air, respectively. The position of the balloons was confirmed with repeated sniffs until a positive deflection in gastric pressure was observed for the gastric catheter, and the esophageal catheter was withdrawn until a negative deflection in P_e_ was observed. An occlusion test was then performed to confirm the catheters’ location in the esophagus (Baydur et al., 1982). P_di_ was calculated online by subtracting P_e_ from gastric pressure. P_di_ and P_e_ were integrated over the period of inspiratory flow and multiplied by breathing frequency and labeled the diaphragm pressure–time product (PTP_di_) and the inspiratory muscle pressure–time product (PTP_e_), respectively. P_di_ and P_e_ were also normalized using the maximum pressure recorded during any maximal inspiratory capacity maneuver performed at rest or during the volitional hyperpnea or control trials for a given experimental visit.

### 2.7 Ventilatory, cardiorespiratory, and perceptual responses

Ventilatory responses during volitional hyperpnea and control trials were measured using a pneumotach (Model 3813; Hans Rudolph, Shawnee Mission, KS, USA) inserted into the mouth port of the two‐way non‐rebreathing valve. Volume was obtained by numerical integration of the flow signal. Operational lung volumes were quantified by measuring inspiratory capacity relative to forced vital capacity. During the resting stage and post‐volitional hyperpnea and control trials, participants performed forced vital capacity and inspiratory capacity maneuvers in triplicate (Guenette et al., 2010). Participants performed further inspiratory capacity maneuvers in duplicate every 2 min (i.e., in the middle of the 2nd, 4th, 6th, 8th, and 10th min) during the volitional hyperpnea and control trials. Strong verbal encouragement was given during each maximal inspiratory effort maneuver. During this time, participants were asked to look forward, minimize any head or neck movement, keep a loose grip on the handlebars, and to avoid talking or swallowing. To confirm that a maximal inspiratory effort was made, we verified that peak inspiratory P_e_ during each inspiratory capacity maneuver matched that obtained at rest. End‐tidal partial pressure of carbon dioxide was measured via the expiratory port of the two‐way nonrebreathing valve, which was connected to a gas analyzer (ML206; AD Instruments, Bella Vista, Australia). Heart rate was measured using the R‐R interval for a three lead ECG (AD Instruments, Bella Vista, Australia) and estimated arterial oxygen saturation was measured using a pulse oximeter (Radical‐7 Pulse CO‐Oximeter; Masimo Corporation, Irvine, CA, USA), respectively. Breathing discomfort, defined as “a feeling of labored or difficult breathing” was measured using the modified 0–10 category ratio Borg Scale (Borg, 1998) during the final min of rest and after every 2 min after the inspiratory capacity maneuvers during volitional hyperpnea and control trials (i.e., at the end of 2nd, 4th, 6th, 8th, and 10th min).

### 2.8 Data capture and analysis

Raw data were sampled using a 16‐channel analogue‐to‐digital data acquisition system (PowerLab 16/35; AD Instruments, Bella Vista, Australia) at 200 Hz. Data was recorded using LabChart v8.1.2 software (AD Instruments, Bella Vista, Australia). Non‐physiological data that resulted from swallowing, coughing, and breath holding were identified by visual inspection and removed. Respiratory muscle pressure, ventilatory, and cardiorespiratory data were continuously sampled and were analyzed in 1 min epochs. These were in the final min of rest and the 2nd (1–2 min), 4th (3–4 min), 6th (5–6 min), 8th (7–8 min), and 10th (9–10 min) min of volitional hyperpnea and control trials.

### 2.9 Blood sampling and enzyme‐linked immunosorbent assays

Ten mL of venous blood was sampled and collected at each time point from an antecubital vein via a BD Vacutainer Winged Blood Collection Set (BD Vacutainer® Safety‐Lok™; Franklin Lakes, NJ, USA) into serum separator tubes (BD Vacutainer® SST™ Tubes; Franklin Lakes, NJ, USA). Samples were centrifuged at 2500 rpm for 10 min. The serum was then aliquoted and stored at −80°C until biochemical assays were performed. Enzyme‐linked immunosorbent assays (ELISA) were performed for serum biomarkers using commercially available kits: Human CKM (Catalog No. ab185988 Abcam, Cambridge, UK); Human Troponin I Type 2, Fast sTnI (Catalog No. RK02421 Abclonal, Woburn MA, USA); and Human Troponin I Type 1, Slow sTnI (Catalog No. RK02420 Abclonal, Woburn MA, USA). ELISAs were performed by following manufacturers' instructions for each specific kit. The assays had detection limits of 30 U/mL (CKM), 17.59 pg/mL (Slow sTnI), and 30 pg/mL (Fast sTnI). To minimize the effect of inter‐assay variation, markers from both volitional hyperpnea and control trials were measured using the same assay plate.

### 2.10 Statistical analysis

Statistical analyses were performed using SPSS 25 for Windows (IBM, Chicago, IL, USA). An initial power calculation was performed on the basis of our previous work (Iqbal et al., 2023), showing that six participants would be required to demonstrate a 10% increase in sTnI with an alpha of 0.05. Normality of the data was assessed by visual inspection of histograms. The data from the volitional hyperpnea and control trials were analyzed using a two‐way analysis of variance (ANOVA) to determine the effects of “time” (rest, 2, 4, 6, 8, and 10 min) for ventilatory, cardiorespiratory, perceptual, and pressure responses or blood analyses (baseline, +1 h, +24 h, and +48 h) and “trial” (volitional hyperpnea vs. control). Following significant interaction effects, pairwise comparisons were made using the Bonferroni method (adjustments for multiple comparisons). Statistical significance was set at *p* ≤ 0.05. Results are presented as means ± SD.

## 3 RESULTS

### 3.1 Ventilatory, cardiorespiratory, perceptual, and pressure responses

**FIGURE 2:**
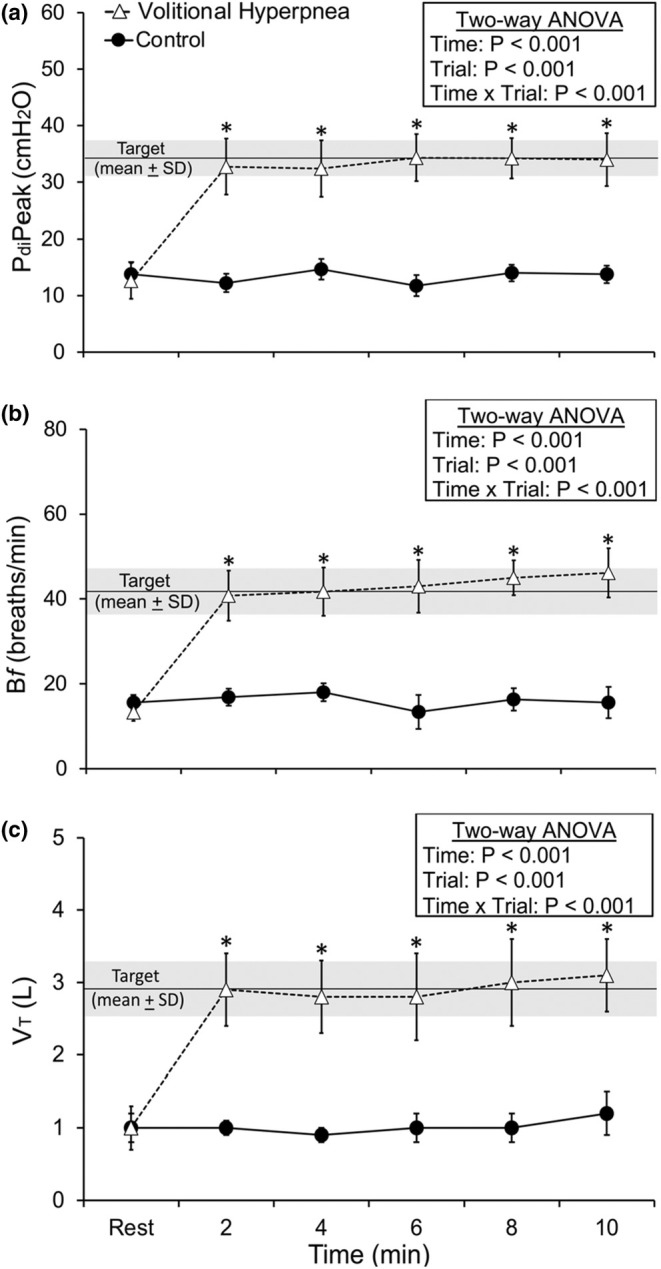
Peak transdiaphragmatic pressure (P_di_Peak; a), breathing frequency (B*f*; b), and tidal volume (V_T_; c) responses to the volitional hyperpnea and control trials. Values are means ± SD. The horizontal black line denotes the mean volitional hyperpnea target and the shaded area the SD of the target. *Significantly different between volitional hyperpnea and control trials (*p* < 0.05; *n* = 8, men).

**FIGURE 3:**
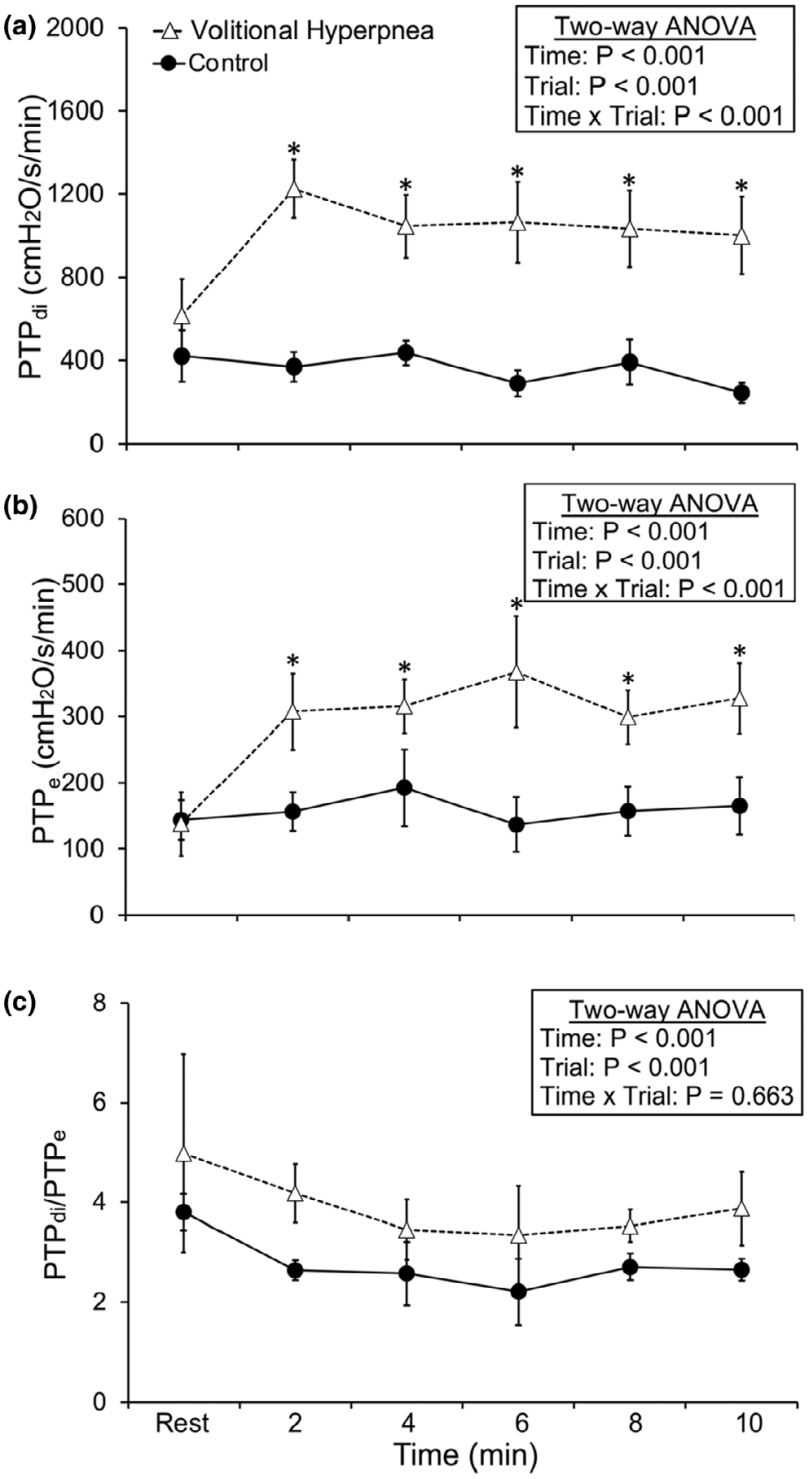
Transdiaphragmatic pressure time product (PTP_di_; a), esophageal pressure time product (PTP_e_; b) and the ratio between PTP_di_ and PTP_e_ (PTP_di_/PTP_e_; c) responses to the volitional hyperpnea and control trials. Values are means ± SD. *Significantly different between volitional hyperpnea and control trials (*p* < 0.05; *n* = 8, men).

The ventilatory, cardiorespiratory, perceptual, and pressure responses at rest and during the volitional hyperpnea and control trials are shown in Table 2 and Figures 2 and 3, along with the main and interaction effects. Significant time × trial interaction effects (*p* < 0.05) were found for minute ventilation, duty cycle, absolute end‐inspiratory lung volume, and as a percentage of forced vital capacity, heart rate, rating of perceived dyspnea, and P_e_ and P_di_ as a percentage of maximum. These responses were significantly higher during volitional hyperpnea compared to the control trial (all *p* < 0.05; Table 2). Time × trial interaction effects (*p* < 0.05) were also observed for peak P_di_, breathing frequency, and tidal volume. These responses were significantly higher at each time point during volitional hyperpnea compared to the control trial (all *p* < 0.05; Figure 2). Time × trial interaction effects (*p* < 0.05) were also observed for PTP_di_ and PTP_e_, but not for PTP_di_/PTP_e_. These responses were significantly higher during volitional hyperpnea compared to the control trial (all *p* < 0.05; Figure 3). Significant time × trial (*p* = 0.01) interaction effects were found for maximal P_di_ measured during the maximal inspiratory capacity maneuvers before and at the end of the volitional hyperpnea and control trials. There was a decrease in maximal P_di_ after the volitional hyperpnea trial (pre: 72.1 ± 20.0 vs. post: 52.1 ± 21.7 cmH_2_O, *p* = 0.05) while no changes were observed in maximal P_di_ after the control trial (pre: 70.1 ± 5.9 vs. post 71.4 ± 9.9 cmH_2_O, *p* = 0.63). These values provide indirect evidence of diaphragm fatigue after the volitional hyperpnea trial. There were no time × trial interaction effects for absolute end‐expiratory lung volume and as a percentage of forced vital capacity, partial pressure of end‐tidal carbon dioxide, and estimated arterial oxygen saturation (Table 2).

**TABLE 2:** Ventilatory, cardiorespiratory, perceptual, and pressure responses to the volitional hyperpnea and control trials. Values are mean ± SD.

Variable	Trial	Rest	2 min	4 min	6 min	8 min	10 min	Time	Trial	Time × trial
V̇_E_ (L/min)	VH	14.3 ± 4.8	119.6 ± 32.6*	118.3 ± 25.2*	116.2 ± 24*	130.5 ± 30.9*	123 ± 36*	<0.001	<0.001	<0.001
	Control	16.6 ± 2.8	16.0 ± 2.5	16.6 ± 3.0	13.6 ± 2.9	16.4 ± 3	15.1 ± 3.1			
Ti/ToT	VH	0.39 ± 0.05	0.50 ± 0.04*	0.50 ± 4*	0.49 ± 0.04*	0.47 ± 0.4*	0.45 ± 0.05*	<0.001	0.015	<0.001
	Control	0.41 ± 0.05	0.41 ± 0.04	0.41 ± 5	0.40 ± 0.05	0.42 ± 0.5	0.41 ± 0.04			
EILV (L)	VH	4.6 ± 0.9	4.9 ± 0.9	4.8 ± 0.9*	5.4 ± 1*	5.8 ± 0.5*	5.6 ± 0.8*	<0.001	0.009	<0.001
	Control	4.6 ± 0.5	4.7 ± 4	4.2 ± 0.7	4.0 ± 0.8	4.3 ± 0.7	4.1 ± 0.6			
EILV (%FVC)	VH	69 ± 11	95 ± 12*	93 ± 10*	94 ± 13*	95 ± 15*	89 ± 14*	<0.001	0.002	<0.001
	Control	69 ± 10	70 ± 12	68 ± 12	67 ± 13	71 ± 12	69 ± 10			
EELV (L)	VH	2.7 ± 0.7	3.4 ± 0.3	3.1 ± 0.3	3.4 ± 0.3	3.0 ± 0.3	3.0 ± 0.4	0.314	0.508	0.117
	Control	3.5 ± 0.4	3.6 ± 0.3	3.2 ± 0.6	2.9 ± 0.8	3.2 ± 0.6	3.1 ± 0.7			
EELV (%FVC)	VH	52 ± 9	55 ± 7	53 ± 5	56 ± 6	53 ± 5	54 ± 7	0.556	0.313	0.237
	Control	60 ± 6	63 ± 6	58 ± 10	53 ± 13	57 ± 10	56 ± 12			
P_ET_CO_2_ (mmHg)	VH	35.3 ± 4.6	36.0 ± 3.2	37.1 ± 3.2	37.8 ± 3.1	38.5 ± 3.0	35.3 ± 4.4	0.021	0.814	0.070
	Control	37.0 ± 3.5	35.8 ± 3.0	37.4 ± 3.2	34.2 ± 4.5	37.8 ± 3.3	35.5 ± 2.8			
SaO_2_ (%)	VH	97.1 ± 1.3	97.7 ± 1.4	97.0 ± 1.6	96.7 ± 1.6	96.0 ± 1.1	96.7 ± 1.3	0.835	1.000	0.089
	Control	96.7 ± 1.8	96.6 ± 1.4	97.7 ± 1.4	97.4 ± 1.3	96.6 ± 1.3	97.4 ± 1.3			
HR (beats/min)	VH	69 ± 11	87 ± 11*	83 ± 9*	82 ± 7*	85 ± 8*	77 ± 9*	<0.001	0.070	<0.001
	Control	70 ± 8	73 ± 9	71 ± 10	66 ± 11	74 ± 10	71 ± 10			
RPD	VH	0.4 ± 0.4	5.4 ± 0.6*	6.9 ± 1.1*	7.3 ± 1*	8.1 ± 1*	8.2 ± 1.7*	<0.001	<0.001	<0.001
	Control	0.4 ± 0.4	0.6 ± 0.3	0.6 ± 0.2	0.4 ± 0.3	0.5 ± 0.3	0.4 ± 0.3			
P_e_ (% maximum)	VH	29 ± 7.5	78 ± 26*	79 ± 24*	79 ± 13*	72 ± 18*	75 ± 21*	<0.001	<0.001	<0.001
	Control	35 ± 7.5	32 ± 6	37 ± 9	30 ± 7.8	36 ± 8.6	38 ± 8.5			
P_di_ (% maximum)	VH	31 ± 5	73 ± 11*	72 ± 12*	76 ± 9*	74 ± 10*	75 ± 11*	<0.001	<0.001	<0.001
	Control	27 ± 7	27 ± 4	33 ± 4	26 ± 4	31 ± 3	31 ± 4			

*Note*: P_e_ is a negative number, but for clarity it has been provided as a positive.

Abbreviations: % maximum, the pressure swing as a % of maximum recorded pressure; EELV, end‐expiratory lung volume; EILV, end‐inspiratory lung volume; FVC, forced vital capacity; HR, heart rate; P_di_, transdiaphragmatic pressure; P_e_, esophageal pressure; P_ET_CO_2_, partial pressure of end‐tidal carbon dioxide; RPD, rating of perceived dyspnea; SaO_2_, estimated arterial oxygen saturation; Ti/ToT, duty cycle; V̇_E_, minute ventilation; VH, volitional hyperpnea.

* Significantly different between volitional hyperpnea and control trials (*p* < 0.05).

### 3.2 Respiratory muscle damage biomarkers

**FIGURE 4:**
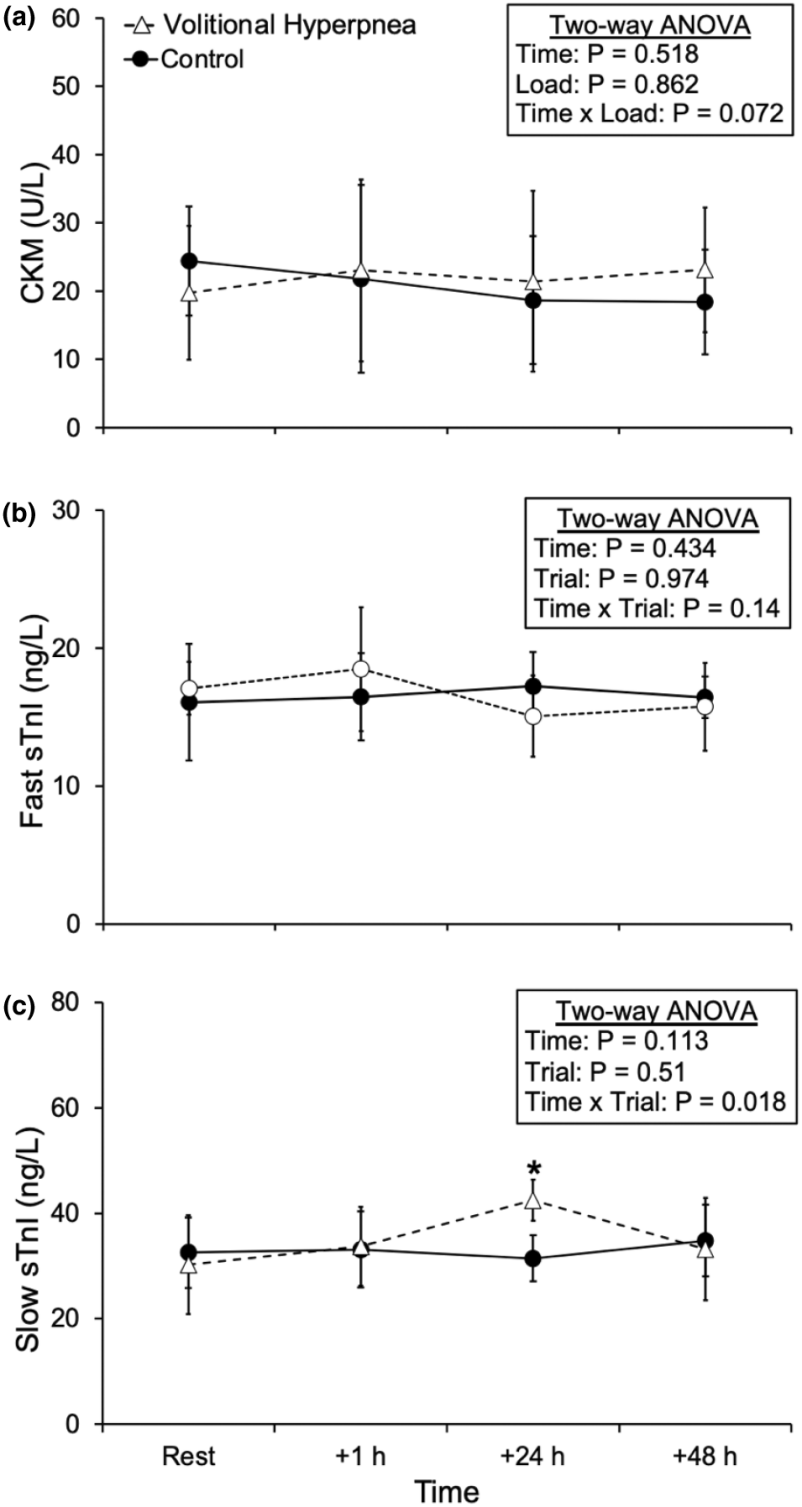
Creatine kinase muscle‐type (CKM; a), fast skeletal troponin I (Fast sTnI; b), and slow skeletal troponin I (Slow sTnI; c) responses to the hyperpnea and control trials. Values are means ± SD. *Significantly different between the volitional hyperpnea and control trials (*p* < 0.05; *n* = 8, men).

The respiratory muscle damage biomarker responses at rest and after volitional hyperpnea and control trials are shown in Figure 4. There was a time × trial interaction effect for slow sTnI only (*p* = 0.018), which was higher compared to the control trial at +24 h (*p* < 0.01) post‐volitional hyperpnea. There were no differences observed at +1 h and +48 h post‐volitional hyperpnea compared with the control trial for slow sTnI. There were no time × trial interaction effects for CKM (*p* = 0.072) and fast sTnI (*p* = 0.14).

## 4 DISCUSSION

### 4.1 Main findings

We investigated the response of a potential panel of serum biomarkers, consisting of CKM and fast and slow sTnI, to detect the potential presence of respiratory muscle damage in response to volitional hyperpnea and control trials undertaken on separate occasions in healthy young men. The main finding was that only slow sTnI was higher at +24 h post‐volitional hyperpnea compared to the same timepoint after the control trial. CKM and fast sTnI did not increase after volitional hyperpnea compared with the control trial. These results suggest that respiratory muscle damage may be present at +24 h following volitional hyperpnea based upon our serum slow sTnI findings, but not evidenced by our serum CKM and fast sTnI findings.

### 4.2 Respiratory muscle damage biomarkers

To our knowledge, we are the first to report previously investigated and recommended respiratory muscle damage biomarkers, CKM, fast sTnI, and slow sTnI in healthy young men in response to volitional hyperpnea that mimicked the breathing (tidal volume, breathing frequency and duty cycle) and respiratory muscle recruitment (P_di_) patterns achieved during high‐intensity exercise. sTnI is a regulatory protein that plays an essential role in the contraction of skeletal muscles (Bowman & Lindert, 2019; Gresslien & Agewall, 2016). We observed that slow sTnI was higher at +24 h post‐volitional hyperpnea compared to the same timepoint after the control trial. These findings align with the outcomes of our previous study where healthy young men performed 60 min of ITL at a resistance equivalent to 70% of their maximal inspiratory mouth pressure, and serum was collected at the same time points as the present study (+1 h, + 24 h, and +48 h) (Iqbal et al., 2023). We found in that study that slow sTnI increased at +24 h and +48 h post‐ITL and is also in agreement with Foster et al. (2012) who found that slow sTnI was elevated by 24% 4 days post‐ITL also at a resistance equivalent to 70% of maximal inspiratory mouth pressure. sTnI exhibits the characteristics of an optimal indicator for skeletal muscle damage, including exclusive specificity to skeletal muscle, a wide diagnostic timeframe that enables early (within 1–6 h after onset) and late (after 24–48 h) detection, and a high level of sensitivity with a notable response magnitude (Sorichter et al., 1997). Our current study supports the conclusions made by Foster et al. (2012) that sTnI has superior sensitivity compared to other non‐specific biomarkers or indices of respiratory muscle damage.

We did not observe an increase in CKM and fast sTnI at any time point post‐volitional hyperpnea compared to the control trial. This is in contrast to our previous finding using ITL (Iqbal et al., 2023), where we observed that CKM and fast sTnI increased within 1 h of ITL, and CKM was increased after 24 and 48 h of ITL. The possible explanation for this finding may be the way the respiratory muscles are loaded during ITL and volitional hyperpnea. Volitional hyperpnea requires more overactivity of the respiratory muscles rather than the overload that is observed in ITL (see *mechanisms of respiratory muscle damage to volitional hyperpnea* below). Alternatively, our volitional hyperpnea protocol may not have been at a sufficient intensity and/or duration to cause respiratory muscle damage because the degree of exertion was lower compared with ITL. Volitional hyperpnea may underestimate the respiratory muscle damage that may occur during whole‐body exercise. For example, Babcock et al. (1995) reported that the workload of the diaphragm needs to be 60%–80% higher during volitional hyperpnea compared to whole‐body exercise to achieve similar levels of diaphragm fatigue. Two potential contributors to diaphragm fatigue during high‐intensity whole‐body exercise are the elevated levels of circulating metabolites from fatiguing peripheral muscles and compromised blood flow to the diaphragm. Given that these two factors are absent during isolated overactivity of the respiratory muscles (i.e., during volitional hyperpnea), respiratory muscle damage might be less than what is expected during whole‐body exercise. This possibly resulted in an insignificant increase in respiratory muscle damage biomarkers (fast sTnI and CKM) in our study. Another possible explanation may be that CKM and fast sTnI are not sensitive enough to measure low‐grade damage caused by volitional hyperpnea as compared to slow sTnI. Further studies utilizing volitional hyperpnea at differing intensities and durations to induce isolated respiratory muscle damage could clarify the relative sensitivities of these serum biomarkers to measure respiratory muscle damage.

### 4.3 Mechanisms of respiratory muscle damage to volitional hyperpnea

Excessive loading that surpasses the normal demands of the respiratory muscles might lead to muscular damage, both during and after the activity. Excessive loading can be classified into two categories – overload or overactivity (Stauber & Smith, 1998). Overload is a condition when the force requirement is higher than usual, while overactivity can be defined as an increased work rate when motor neurons fire much higher than typically experienced during physiological duty cycles (Stauber & Smith, 1998). Overactivity occurs during many endurance exercise activities and in several disease conditions such as asthma or chronic obstructive pulmonary disease (Stauber & Smith, 1998). In respiratory diseases, the respiratory muscles can undergo both overload and overactivity due to the increased demand for inspiratory muscle force and breathing frequency (Stauber & Smith, 1998). In the present study, volitional hyperpnea was used to induce a similar kind of overactivity of respiratory muscles by voluntarily increasing breathing frequency and tidal volume. This exceeded the respiratory muscles usual capability, and this overactivity was aimed at causing damage to the muscles involved in breathing (Foster et al., 2012; Mathur et al., 2010). Volitional hyperpnea‐induced respiratory muscle damage may specifically affect a specific number of components in the muscle cells, or it could cause minor tears in various components such as the sarcolemma, z‐disk, basal lamina, and surrounding connective tissues. Additionally, it can lead to damage in the cytoskeleton and contractile elements (Fridén & Lieber, 1996; Lieber et al., 1985; Roth et al., 1999, 2000). In the current study, we successfully detected a small but significant increase in the concentrations of slow sTnI in the participants' serum, suggesting the presence of respiratory muscle damage after volitional hyperpnea. Fast and slow sTnI correspond to fast and slow twitch muscle fibers, respectively. The most accurate estimates of fiber type distribution in the adult human diaphragm suggest approximately 55% slow, 21% fast oxidative, and 24% fast glycolytic fibers (Polla et al., 2004; Smith‐Blair, 2002). The proportion of slow twitch fibers exceeds 60% in both the internal and external intercostal muscles, slightly higher than in the diaphragm (Polla et al., 2004; Smith‐Blair, 2002). This differential proportion of fiber types in the human respiratory muscles may also have resulted in a differential release of sTnI isoforms in serum following the volitional hyperpnea trial. The high percentage of slow twitch muscle fibers in the inspiratory muscles means that they may be preferentially damaged over fast twitch muscle fibers.

### 4.4 Limitations

Our study presents three limitations. Firstly, males only were studied. The purpose of this study was to elucidate the effects of volitional hyperpnea on biomarkers of respiratory muscle damage rather than to address potential sex‐based differences which, however, we recognize are present in respiratory physiology (Dominelli & Molgat‐Seon, 2022). Therefore, future investigations should explore the impact of sex on changes in respiratory muscle damage biomarkers following volitional hyperpnea. Secondly, due to logistical constraints, serum samples were exclusively collected at 1, 24, and 48 h after volitional hyperpnea. While these time points are suitable enough for detecting respiratory muscle damage, it is recommended that future research examine these biomarkers at additional intervals within the 24‐h period. This approach would provide a more comprehensive understanding of the changes in these biomarkers in response to respiratory muscle damage. Finally, our sample size raises the possibility of type II errors. This occurred due to challenges in recruitment, primarily due to the time commitments, perceived invasiveness of testing, and restrictions on human testing due to COVID‐19.

## 5 CONCLUSION

We investigated the response of a potential panel of serum biomarkers, consisting of CKM and fast and slow sTnI, to detect the presence of respiratory muscle damage in response to volitional hyperpnea and control trials undertaken on separate occasions in healthy young men. The main finding was that only slow sTnI was higher at +24 h post volitional hyperpnea compared to the same timepoint after the control trial. CKM and fast sTnI did not increase after volitional hyperpnea compared with the control trial. These results suggest that respiratory muscle damage may have been present at +24 h following volitional hyperpnea based upon our serum slow sTnI findings but not evidenced by our serum CKM and fast sTnI findings. Future studies including more timepoints, different respiratory muscle exercise protocols, and examining the differences between sexes could provide additional insights into the utility of blood biomarkers for identifying respiratory muscle damage.

## AUTHOR CONTRIBUTIONS

E.W., B.H., D.E.M. conceived and designed the experiments; M.I., E.B., D.E.M. performed the experiments; M.I. analyzed the data; M.I., D.E.M. wrote the paper. E.B., E.W., B.H., D.E.M. reviewed the paper.

## FUNDING INFORMATION

This research was funded by the UniSQ International Research Scholarship, University of Southern Queensland.

## CONFLICT OF INTEREST STATEMENT

The authors declare no conflict of interest.

## ETHICS STATEMENT

All study procedures were approved by the University of Southern Queensland Human Research Ethics Committee (H20REA151), which adheres to the Declaration of Helsinki with the exception of registration in a database.
